# A Cystine-Rich Whey Supplement (Immunocal®) Provides Neuroprotection from Diverse Oxidative Stress-Inducing Agents *In Vitro* by Preserving Cellular Glutathione

**DOI:** 10.1155/2017/3103272

**Published:** 2017-08-15

**Authors:** Aimee N. Winter, Erika K. Ross, Vamsi Daliparthi, Whitney A. Sumner, Danielle M. Kirchhof, Evan Manning, Heather M. Wilkins, Daniel A. Linseman

**Affiliations:** ^1^Department of Biological Sciences, University of Denver, 2199 S. University Blvd., Denver, CO 80208, USA; ^2^Knoebel Institute for Healthy Aging, University of Denver, 2155 E. Wesley Ave., Denver, CO 80208, USA

## Abstract

Oxidative stress is a principal mechanism underlying the pathophysiology of neurodegeneration. Therefore, nutritional enhancement of endogenous antioxidant defenses may represent a viable treatment option. We investigated the neuroprotective properties of a unique whey protein supplement (Immunocal®) that provides an essential precursor (cystine) for synthesis of the endogenous antioxidant, glutathione (GSH). Primary cultures of rat cerebellar granule neurons (CGNs), NSC34 motor neuronal cells, or HT22 hippocampal cells were preincubated in medium containing Immunocal and then subsequently treated with agents known to induce oxidative stress. Immunocal protected CGNs against neurotoxicity induced by the Bcl-2 inhibitor, HA14-1, the nitric oxide donor, sodium nitroprusside, CuCl_2_, and AlCl_3_. Immunocal also significantly reduced NSC34 cell death due to either H_2_O_2_ or glutamate and mitigated toxicity in HT22 cells overexpressing *β*-amyloid_1-42_. The neuroprotective effects of Immunocal were blocked by inhibition of *γ*-glutamyl-cysteine ligase, demonstrating dependence on de novo GSH synthesis. These findings indicate that sustaining GSH with Immunocal significantly protects neurons against diverse inducers of oxidative stress. Thus, Immunocal is a nutritional supplement worthy of testing in preclinical animal models of neurodegeneration and in future clinical trials of patients afflicted by these diseases.

## 1. Introduction

Oxidative stress and mitochondrial dysfunction are major factors underlying the pathophysiology of several neurodegenerative disorders including Parkinson's disease, Alzheimer's disease, and amyotrophic lateral sclerosis (ALS) [1–4]. For instance, complex I deficiency and the consequent increase in mitochondrial reactive oxygen species (ROS) play a critical role in the death of dopaminergic neurons in Parkinson's disease [5, 6]. In models of Alzheimer's disease, evidence of mitochondrial dysfunction and oxidative stress precedes the deposition of characteristic amyloid beta-plaques during disease progression [7, 8]. In the case of ALS, mutant forms of copper-zinc superoxide dismutase (SOD1), which are collectively responsible for approximately 20% of cases of familial ALS, accumulate at mitochondria and trigger a shift in the redox state of these organelles [9]. The above findings strongly indicate that oxidative stress, particularly at the level of the mitochondria, plays a central role in the neuronal death that underlies a diverse group of neurodegenerative diseases.

Glutathione (GSH) is an endogenous tripeptide antioxidant that plays a key role in preventing oxidative stress, thereby preserving mitochondrial function and averting cellular apoptosis [10]. In many neurodegenerative disorders, GSH levels have been shown to be significantly depleted in patients suffering from these diseases, resulting in a diminished capacity to cope with increases in cellular ROS [11–13]. Indeed, decreases in GSH are often observed to precede other hallmarks of disease pathology, such as complex I deficiency and loss of dopaminergic neurons in Parkinson's disease [14]. Intriguingly, *in vitro* studies on GSH depletion have demonstrated that decreases in total cellular GSH levels can recapitulate disease pathology. For instance, in a dopaminergic PC12 cell line, deficiencies in GSH synthesis that led to an overall decrease in cellular GSH resulted in complex I inhibition, increased indices of oxidative stress, and deficits in mitochondrial respiration, as seen in cases of Parkinsonism [15]. Similarly, NSC34 motor neuron-like cells stably expressing the human G93A mutant form of SOD1 displayed a significant and selective depletion of mitochondrial GSH content in comparison to parental cells, reminiscent of some forms of familial ALS [16]. GSH depletion *in vitro* has also been shown to sensitize neurons to oxidative stress and mitochondrial dysfunction, leading to subsequent increases in ROS and apoptotic cell death. This was clearly demonstrated by a study in which primary cortical neurons treated with subtoxic levels of the GSH-depleting agent, buthionine sulfoximine (BSO), underwent apoptosis in the presence of trace amounts of extracellular copper [17]. Similarly, TAR DNA-binding protein-43 (TDP-43) forms cytoplasmic inclusions, which are a hallmark pathology observed in sporadic ALS patients, in cultured neurons subjected to GSH depletion [18]. Collectively, these studies demonstrate a critical role for GSH depletion in disease progression and pathology in multiple neurodegenerative disease states.

Given the prominent relationship between GSH depletion and neurodegeneration, it is not surprising that many studies have been undertaken to determine the neuroprotective effects of bolstering GSH levels through various treatment paradigms. Such treatments include administration of the GSH precursor, N-acetylcysteine (NAC), and GSH-monoethylester (GSH-MEE), a cell permeable form of GSH, and induction of the transcription factor, nuclear factor erythroid 2-related factor-2 (Nrf2), which is involved in transcriptional regulation of *γ*-glutamyl-cysteine ligase, the rate-limiting enzyme necessary for GSH synthesis [19]. Studies with NAC are extensive and indicate that NAC treatment offers a number of benefits across numerous disease models. For example, NAC demonstrated a significant protective capacity in a rotenone (complex I inhibition) rat model of Parkinson's disease by decreasing ROS generation, sustaining normal GSH levels, and ultimately preventing dopaminergic cell death [20]. In the G93A mutant SOD1 mouse model of familial ALS, NAC delayed the onset of disease-associated motor deficits and significantly extended survival, possibly due to its ability to elevate GSH levels in these animals [21]. Lastly, SAMP8 senescence-accelerated mice, which display many of the pathological features of Alzheimer's disease, demonstrated an increased cognitive performance with NAC treatment as compared to vehicle-treated controls [22]. Another study utilizing GSH-MEE in an MPTP rat model of Parkinson's disease demonstrated that GSH-MEE supplementation is capable of raising GSH levels in the brain when centrally delivered, and this increase in GSH corresponded to partial preservation of striatal dopamine levels [23]. Studies such as this have led to recent clinical trials testing the safety and tolerability of intranasal delivery of GSH to patients with PD [24]. Finally, Nrf2 induction or overexpression has shown similar promise in animal models of Parkinson's, ALS, and Alzheimer's disease. In the MPTP mouse model of Parkinson's disease, overexpression of Nrf2 in astrocytes attenuated the development of a Parkinsonian phenotype [25]. Likewise, astrocytic overexpression of Nrf2 in a mouse model of ALS both delayed onset and increased survival, as did treatment with chemical Nrf2 inducers [26, 27]. Comparatively, lentiviral Nrf2 overexpression caused significant improvements in observed learning deficits in a mouse model of Alzheimer's disease, accompanied by decreased amyloid plaque burden [28]. Cumulatively, these data indicate that treatments aimed at increasing GSH levels in the brain may be a viable option for treatment and prevention of neurodegenerative disease.

However, while existing treatment strategies have shown some promise in this capacity, the efficacy of such treatments is significantly limited by the relatively low stability and bioavailability of compounds such as GSH-MEE and NAC [23, 29]. Moreover, GSH-MEE requires direct injection into the brain for significant effects to be observed, further limiting its efficacy for treatment in human patients [23]. In the current study, we investigated the neuroprotective potential of a nondenatured whey protein supplement, Immunocal, *in vitro* in several models of oxidative stress. Immunocal has previously been shown to substantially increase blood or lymphocyte GSH levels in patients with HIV infection or cystic fibrosis, respectively, owing to its high concentration of nondenatured whey proteins containing the cysteine precursor, cystine (see Table 1 for composition) [30–32]. Cystine is resistant to trypsin proteolysis and able to travel through the bloodstream to the target cell where it is then readily reduced to two cysteine molecules which can serve as essential precursors for de novo GSH synthesis. In this manner, the stability of Immunocal lends itself to increased bioavailability, such that it can act as a cysteine delivery system. This is significant, as cysteine is spontaneously catabolized in the GI tract and bloodstream, and its supplementation alone can produce toxicity [33]. Additionally, because of its superior stability, the effects of Immunocal are not dependent upon an invasive administration system as is needed for GSH-MEE and have been observed with standard oral dosing regimens. These unique characteristics spurred us to examine the neuroprotective potential of Immunocal.

## 2. Materials and Methods

### 2.1. Materials

Immunocal was provided by Immunotec Inc. (Quebec, Canada; Table 1). 2-Amino-6-bromo-*α*-cyano-3-(ethoxycarbonyl)-4H-1-benzopyran-4-acetic acid ethyl ester (HA14-1) and sodium nitroprusside (SNP) were obtained from Calbiochem (San Diego, CA). DL-buthionine-sulfoximine (BSO), 4, 6-diamidino-2-phenylindole (DAPI), Hoechst dye 33258, and a monoclonal antibody against *β*-tubulin (clone AA2; used at a dilution of 1 : 250) were from Sigma Aldrich Co. LLC (St Louis, MO). FITC-conjugated secondary antibodies were from Jackson Immunoresearch Laboratories (West Grove, PA).

### 2.2. Cell Culture and Treatment

Rat cerebellar granule neurons (CGNs) were isolated as previously described from 7-day-old Sprague-Dawley rat pups of both sexes [34]. CGNs were seeded on 35 mm diameter plastic dishes coated with poly-L-lysine at an average density of 2.0 × 10^6^ cells/mL in basal modified Eagle's medium containing 10% fetal bovine serum, 25 mM KCl, 2 mM L-glutamine, and penicillin (100 units/mL)/streptomycin (100 *μ*g/mL). Cytosine arabinoside (10 *μ*M) was added to the culture medium 24 h after plating. Experiments were performed after 6 days in culture. In general, cells were pretreated with Immunocal at a concentration of 3.3%, *w*/*v* (unless otherwise noted) in serum-free medium for 24 h prior to treatment with the specified insult (i.e., SNP, HA14-1, etc.) for an additional 24 h.

NSC34 cells were maintained in DMEM with high glucose containing 10% fetal bovine serum, 2 mM L-glutamine, and penicillin (100 units/mL)/streptomycin (100 *μ*g/mL). NSC34 cells were preincubated with Immunocal for 24 h prior to exposure to H_2_O_2_ or glutamate. For glutamate experiments, NSC34 cells were differentiated by withdrawing serum for 7 days prior to experimentation.

For transient transfection, HT22 mouse hippocampal cells were seeded in 6-well plates at an approximate confluency of 1.0 × 10^6^ cells/mL and then cultured for 24 h in DMEM with low glucose containing 10% fetal bovine serum, 2 mM L-glutamine, and penicillin (100 units/mL)/streptomycin (100 *μ*g/mL). Cells were transfected using lipofection (5 *μ*g DNA/mL, 5 *μ*L lipofectamine/mL) in OptiMEM medium for 4 h with either empty pIRES 2DsRed-Express2 bicistronic vector (Clontech, Mountain View, CA) or vector containing the sequence for amyloid-beta 1-42 (A*β*_1-42_). Following transfection, OptiMEM medium was replaced with DMEM culture medium, and cells were treated with Immunocal for 24 h. Percent apoptosis was then determined for only transfected (DSRed-positive) cells based on nuclear morphology.

### 2.3. Cell Viability, Lipid Peroxidation, and Cellular GSH Assay

All assays were performed according to commercially available manufacturer's instructions. GSH/GSSG assay kit was purchased from Oxford Biomedical Research (Oxford, MI). MTT cell viability assay was from BioAssay Systems (Hayward, CA). Malondialdehyde (MDA) lipid peroxidation assay was obtained from OXIS Research Inc. (Foster City, CA).

### 2.4. Immunofluorescence Microscopy

After treatment, cells were fixed in 4% paraformaldehyde for 1 h, washed once in PBS, and then permeabilized and blocked in 0.2% Triton X-100 and 5% bovine serum albumin (BSA) in PBS. Primary antibody (monoclonal antibody against *β*-tubulin; clone AA2; used at a dilution of 1 : 250; Sigma Aldrich Co. LLC, St Louis, MO) was diluted in 2% BSA and 0.2% Triton X-100 in PBS, and cells were incubated with primary antibodies for 24 h at 4°C. They were then washed 5 times in PBS and then incubated for 1 h in FITC-conjugated secondary antibody diluted in 2% BSA and 0.2% Triton-X 100 in PBS with DAPI. The cells were washed 5 times with PBS before the addition of antiquench (0.1% *p*-phenylenediamine in PBS). Images were captured using a Zeiss Axiovert 200 M epifluorescence microscope equipped with Zeiss Axiovision software.

### 2.5. Statistical Analysis

Each experiment was done in duplicate and repeated a minimum of three times; data are reported as mean ± SEM. Statistical significance was analyzed with a one-way analysis of variance (ANOVA) followed by post hoc Tukey's test.

## 3. Results

### 3.1. Immunocal Preserves Cellular GSH and Prevents Apoptosis in CGNs Exposed to the Bcl-2 Inhibitor, HA14-1

Initially, primary CGNs were incubated with 3.3% (*w*/*v*) Immunocal for 24 h to assess any potential toxicity that this supplement might induce. Immunocal is composed of five primary cystine- and glutamylcysteine-containing proteins, *β*-lactoglobulin, immunoglobulin, *α*-lactalbumin, serum albumin, and lactoferrin (Table 2) [35, 36]. Based upon the relative percentages for each of these four proteins within the whey protein fraction and the number of cystine or glutamylcysteine residues contained within each protein, we calculated the approximate concentration of each of these GSH precursors with which CGNs were treated (Table 3). In general, a 3.3% solution of Immunocal in culture medium contains 85.3 mM cystine and 30 mM glutamylcysteine, both of which have the potential to act as GSH precursors; however, it should be noted that since both precursors are contained within much larger proteins it is unlikely that all cystine and glutamylcysteine molecules are freely available to be utilized in GSH synthesis. Thus, the values calculated in Table 3 for these precursors should be considered as concentrations that could potentially be achieved rather than absolute concentrations.

**Figure 1:**
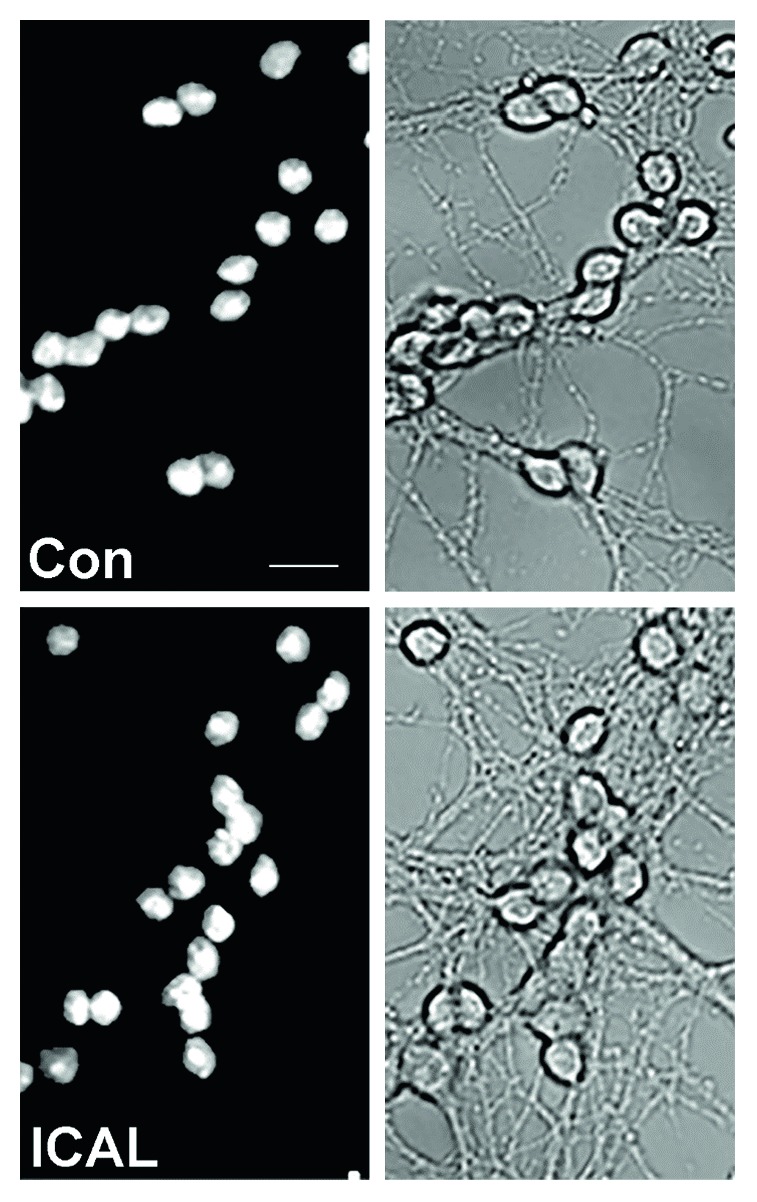
Cells treated with Immunocal display healthy neuronal morphology. Cells were left untreated (a) or treated with Immunocal alone (b) and assessed for overall health and appearance. Left-hand panels are representative images of cell nuclei stained with DAPI. Right-hand panels depict the same fields as viewed under brightfield to assess the state of neuronal processes and soma. Con: control; ICAL: Immunocal. Scale bar, 10 *μ*m.

Following Immunocal treatment, cells were fixed and stained with DAPI to analyze nuclear morphology. Cells treated with Immunocal alone displayed nuclear morphology comparable to that of untreated control cells (Figure 1). Moreover, observation under brightfield demonstrated that cells treated with Immunocal maintained a healthy neuronal morphology with intact processes and large somas, comparable to cells that were not supplemented with Immunocal (Figure 1).

**Figure 2:**
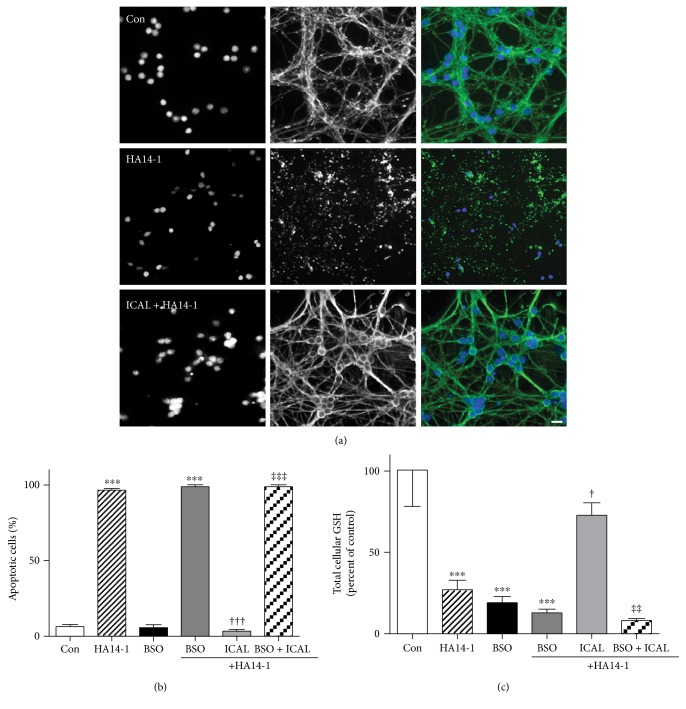
Immunocal preserves cellular GSH and prevents apoptosis in CGNs exposed to the Bcl-2 inhibitor, HA14-1. (a) Representative images of CGNs left untreated (control), treated with HA14-1 (15 *μ*M), or preincubated for 24 h with Immunocal before HA14-1 treatment for further 24 h. Panels from left to right, DAPI (nuclei), *β*-tubulin, merged images showing *β*-tubulin (green), and DAPI (blue). Scale bar, 10 *μ*m. (b) Quantification of apoptosis for 4 independent experiments performed as in (a) except some cultures were preincubated with 200 *μ*M BSO as well. Apoptotic cells were those with condensed or fragmented nuclei. Results are shown as mean ± SEM, *n* = 4. ∗∗∗ indicates *p* < 0.001 compared to control, ††† indicates *p* < 0.001 compared to HA14-1, ‡‡‡ indicates *p* < 0.001 compared to ICAL + HA14-1. (c) CGNs were treated exactly as described in (b). Total cellular GSH was measured as described in Materials and Methods. Data shown represent the percent of control cellular GSH concentration, mean ± SEM, *n* = 4. ∗∗∗ indicates *p* < 0.001 compared to control, † indicates *p* < 0.05 compared to HA14-1, and ‡‡ indicates *p* < 0.01 compared to ICAL + HA14-1. Significant differences were determined by one-way ANOVA with a post hoc Tukey's test. Con: control; ICAL: Immunocal; BSO: buthionine sulfoximine.

Having established that Immunocal displayed no overt toxicity to CGNs, cells were next treated with Immunocal and then exposed to the Bcl-2 homology-3 domain (BH3) mimetic, HA14-1. We have previously shown this Bcl-2 inhibitor to induce GSH-sensitive mitochondrial oxidative stress and intrinsic apoptosis in CGNs [37, 38]. HA14-1 induced marked nuclear condensation and microtubule disruption (Figure 2(a)) indicative of apoptosis (Figure 2(b)), while also causing significant depletion of GSH (Figure 2(c)). Immunocal significantly protected CGNs from apoptosis induced by HA14-1 and significantly preserved GSH levels. To confirm that the mechanism of protection was dependent, at least in part, on enhanced GSH synthesis, CGNs were cotreated with Immunocal and the *γ*-glutamyl-cysteine ligase inhibitor, BSO, which prevents GSH synthesis [39]. Coincubation with Immunocal and BSO for 24 h before HA14-1 treatment completely prevented any protective effect that Immunocal alone displayed against the Bcl-2 inhibitor (Figure 2(b)). Moreover, the capacity of Immunocal to preserve cellular GSH levels upon HA14-1 exposure was eliminated by BSO cotreatment (Figure 2(c)).

### 3.2. Immunocal Protects CGNs from CuCl_2_-Induced Oxidative Damage and Decreases Cellular Lipid Peroxidation

**Figure 3:**
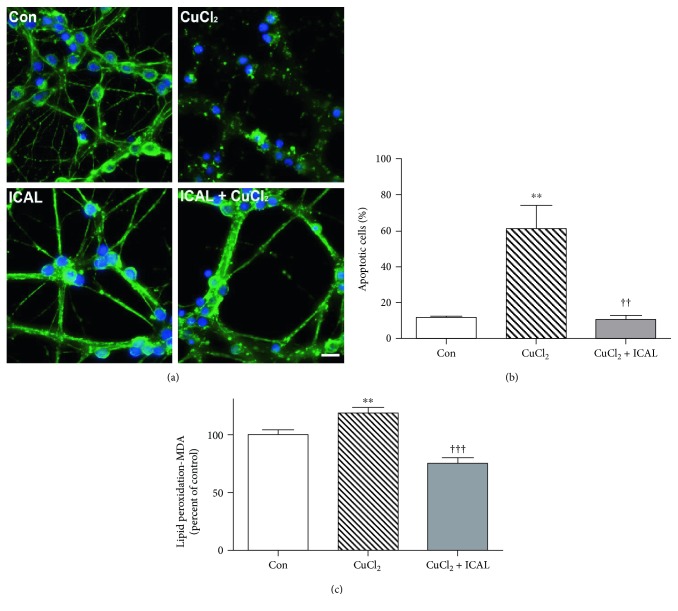
Immunocal decreases CuCl_2_-induced apoptosis and lipid peroxidation in CGNs. (a) Representative images of CGNs left untreated (control), treated with CuCl_2_ (50 *μ*M), or preincubated with Immunocal for 24 h before CuCl_2_ treatment for further 24 h. Immunofluorescence shows *β*-tubulin (green) and DAPI (blue). Scale bar, 10 *μ*m. (b) Quantification of apoptosis for 4 independent experiments performed as in (a). Results are shown as mean ± SEM, *n* = 4. ∗∗ indicates *p* < 0.01 compared to control and †† indicates *p* < 0.01 compared to CuCl_2_. (c) Cellular lipid peroxidation (malondialdehyde (MDA)) was measured as described in Materials and Methods. Results are shown as mean ± SEM, *n* = 5. ∗∗ indicates *p* < 0.01 compared to control, ††† indicates *p* < 0.001 compared to CuCl_2_. Con: control; ICAL: Immunocal.

To further investigate the neuroprotective potential of Immunocal in primary neurons, we used copper chloride (CuCl_2_) as a model of oxidative stress. Copper overload is associated with free radical-induced lipid peroxidation and disruption of mitochondrial complex activity [40, 41]. Immunofluorescence analysis of the microtubule network revealed robust protection from this transition metal in CGNs pretreated with Immunocal (Figure 3(a)). Quantification of apoptotic cells revealed that there was a significant reduction in CGN apoptosis with Immunocal pretreatment compared to CGNs treated with CuCl_2_ alone (Figure 3(b)). The antioxidant effect of Immunocal was confirmed with a lipid peroxidation assay which revealed a significant decrease in malondialdehyde content in CGNs pretreated with Immunocal (Figure 3(c)).

### 3.3. Immunocal Protects CGNs Exposed to Sodium Nitroprusside- (SNP-) Generated Nitric Oxide Species and from AlCl_3_-Induced Neurotoxicity

**Figure 4:**
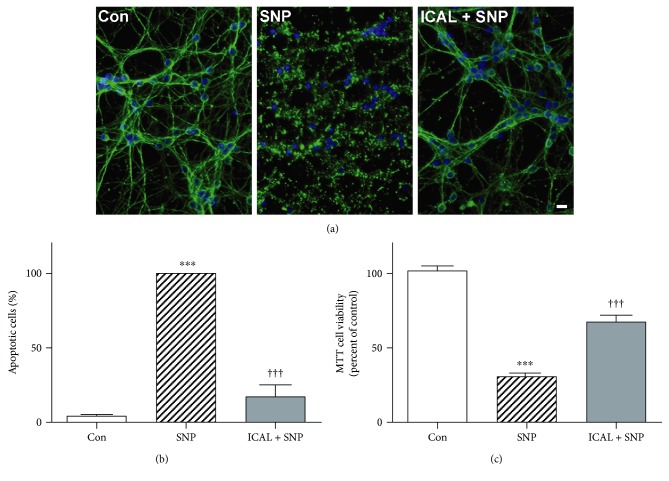
Immunocal preserves CGN viability and protects from apoptosis after exposure to SNP. (a) Representative images of CGNs left untreated (control), treated with SNP (100 *μ*M), or preincubated with Immunocal for 24 h before SNP treatment for further 24 h. Immunofluorescence shows *β*-tubulin (green) and DAPI (blue). Scale bar, 10 *μ*m. (b) Quantification of apoptosis for 5 independent experiments performed as in (a). Results are shown as mean ± SEM, *n* = 5. (c) MTT cell viability was measured as described in Materials and Methods. Results are shown as mean ± SEM, *n* = 3. For (b) and (c), ∗∗∗ indicates *p* < 0.001 compared to control, and ††† indicates *p* < 0.001 compared to SNP. Con: control; ICAL: Immunocal.

SNP is a nitric oxide donor that causes dissipation of the mitochondrial membrane potential and enhanced generation of mitochondrial ROS in cortical neurons and CGNs [42, 43]. As expected, nitric oxide species generated by SNP caused overt apoptotic cell death in CGNs which was significantly mitigated by pretreatment with Immunocal (Figure 4(a)). Apoptotic cell counts confirmed that there was significant neuroprotection in CGNs pretreated with Immunocal, decreasing apoptosis by approximately 80% (Figure 4(b)). An MTT cell viability assay demonstrated similar results and showed that mitochondrial viability was also significantly preserved in Immunocal-pretreated cells, compared to CGNs treated with SNP alone (Figure 4(c)).

**Figure 5:**
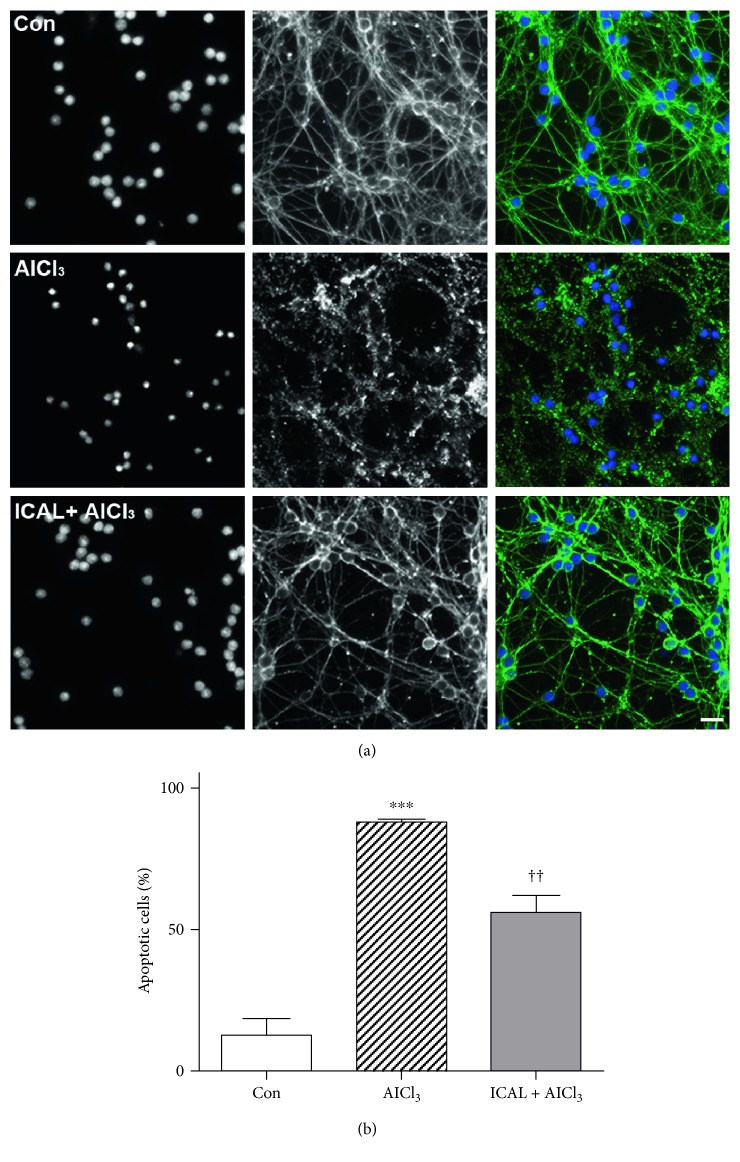
Immunocal protects CGNs from AlCl_3_-induced toxicity. (a) Representative images of CGNs left untreated (control), treated with AlCl_3_ (10 *μ*M), or preincubated with Immunocal for 24 h before AlCl_3_ treatment for further 48 h. Panels from left to right, DAPI (nuclei), *β*-tubulin, and merged image showing *β*-tubulin (green), and DAPI (blue). Scale bar, 10 *μ*m. (b) CGN apoptosis was quantified for 4 independent experiments as described in (a). Results are shown as mean ± SEM, *n* = 4. ∗∗∗ indicates *p* < 0.001 compared to control, and †† indicates *p* < 0.01 compared to AlCl_3_. Con: control; ICAL: Immunocal.

Aluminum is a neurotoxic metal that impairs mitochondrial structure and function in neural cells exposed *in vitro* and *in vivo* [44, 45]. Aluminum chloride- (AlCl_3_-) induced toxicity in CGNs was characterized by nuclear condensation and marked disruption of the microtubule network; these effects were markedly decreased in CGNs pretreated with Immunocal (Figure 5(a)). To confirm that this protection was due to cysteine supplementation, and not metal chelation, we removed the Immunocal after the pretreatment period and washed the CGNs with serum-free media before treating with AlCl_3_. Under these conditions, we still observed a significant reduction in apoptosis compared to CGNs treated with AlCl_3_ alone (Figure 5(b)).

### 3.4. Immunocal Protects NSC34 Motor Neuron-Like Cells from H_2_O_2_ and Glutamate/Glycine-Induced Excitotoxicity

**Figure 6:**
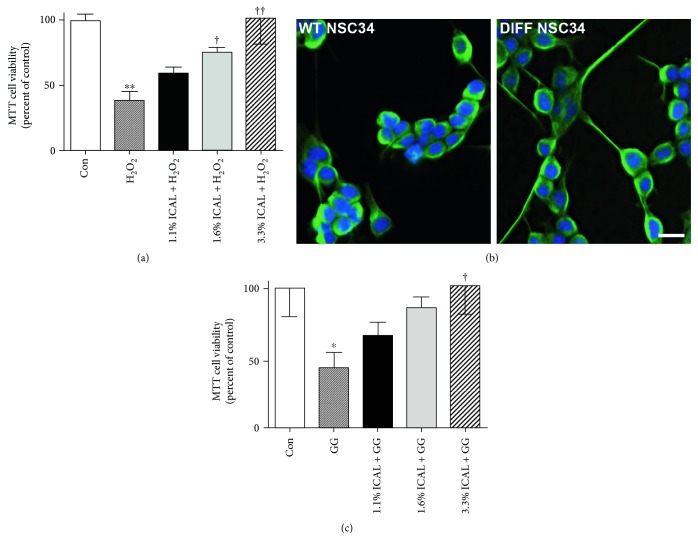
Immunocal protects NSC34 cells from H_2_O_2_ and glutamate/glycine-induced excitotoxicity. (a) Cell survival was quantified with MTT cell viability assay for 5 independent experiments in undifferentiated NSC34 left untreated (control), treated with H_2_O_2_ (250 *μ*M), or preincubated with Immunocal for 24 h before H_2_O_2_ treatment for further 24 h. Results are shown as mean ± SEM, *n* = 5. ∗∗ indicates *p* < 0.01 compared to control, † indicates *p* < 0.05 compared to H_2_O_2_, and †† indicates *p* < 0.01 compared to H_2_O_2_. (b) Representative images showing morphological differences between undifferentiated (wildtype (WT)) and differentiated (DIFF) NSC34 cells, *β*-tubulin (green), and DAPI (blue). Scale bar, 10 *μ*m. (c) Cell survival was quantified for 5 independent experiments with an MTT cell viability assay in differentiated NSC34 cells left untreated (control), treated with glutamate/glycine (1 mM/100 *μ*M), or preincubated with Immunocal for 24 h before glutamate/glycine treatment for further 24 h. ∗ indicates *p* < 0.05 compared to control, and † indicates *p* < 0.05 compared to glutamate/glycine. Con: control; ICAL: Immunocal; GG: glutamate/glycine.

H_2_O_2_-mediated cell death is a classic model of ROS toxicity in neuronal systems, as it generates free radicals that are implicated in neurodegeneration [46]. As expected, ROS generated by H_2_O_2_ caused an overt loss of viability in NSC34 cells, which was significantly mitigated by pretreatment with Immunocal. An MTT cell viability assay demonstrated that mitochondrial viability was preserved in Immunocal-pretreated cells in a dose-dependent manner, compared to NSC34 cells treated with H_2_O_2_ alone (Figure 6(a)). Incubation with Immunocal alone had no significant adverse effect on NSC34 cell viability assessed by MTT assay (data not shown).

Glutamate excitotoxicity is thought to play a significant role in several forms of neurodegenerative disease, leading to neuronal damage and cell death through both apoptotic and nonapoptotic mechanisms. NSC34 motor neuron-like cells do not typically express functional glutamate receptors, which are the primary mediators of excitotoxicity. However, if they are exposed to serum withdrawal for 7 days, then they attain a semi-differentiated state and express functional N-methyl-D-aspartate (NMDA) receptors (Figure 6(b)). After this, point cells become sensitive to glutamate excitotoxicity [47]. We observed that exposure to glutamate/glycine caused a significant loss of viability in NSC34 cells differentiated by serum withdrawal. An MTT cell viability assay demonstrated that mitochondrial viability was significantly preserved in Immunocal-pretreated cells in a dose-dependent manner, compared to NSC34 cells treated with glutamate/glycine alone (Figure 6(c)).

### 3.5. Immunocal Protects HT22 Mouse Hippocampal Cells from Toxicity Induced by Overexpression of Amyloid-Beta Peptide (A*β*_1-42_)

**Figure 7:**
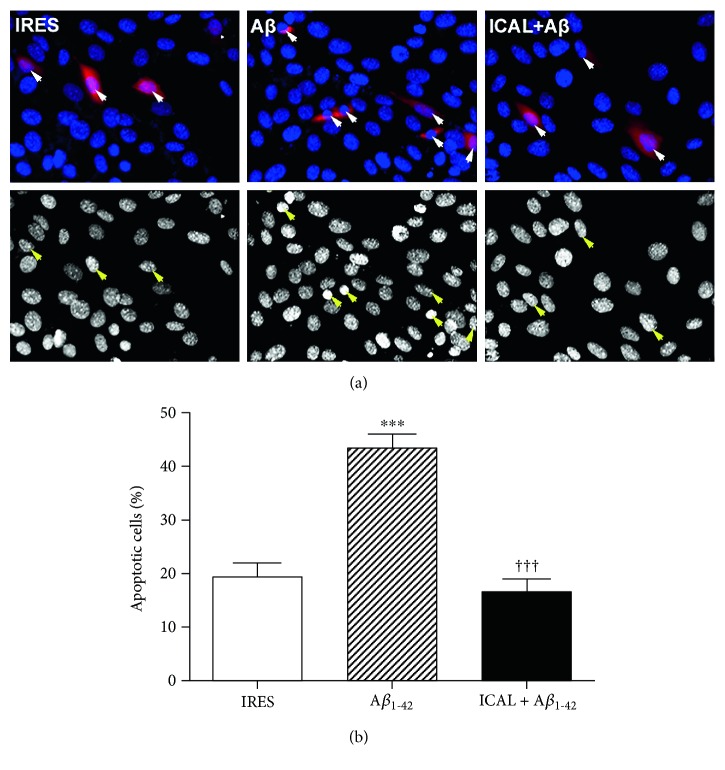
Immunocal protects HT22 cells from toxicity induced by overexpression of A*β*_1-42_. (a) Representative images of HT22 cells transfected with either empty vector (IRES) or A*β*_1-42_. Top panels display colored images showing successful transfection of the cells, and bottom panels display decolorized images of cell nuclei to visualize nuclear condensation. Arrows indicate transfected cells. (b) Quantification of apoptosis for 4 independent experiments performed as in (a). Results are shown as mean ± SEM, *n* = 4. ∗∗∗ indicates *p* < 0.001 compared to control, and ††† indicates *p* < 0.001 compared to cells transfected with A*β*_1-42_ without Immunocal preincubation. A*β*: amyloid-beta; ICAL: Immunocal.

A*β*_1-42_ is the major constituent of senile plaques, which form in the brains of Alzheimer's patients, leading to the hypothesis that increased production of this protein from aberrant processing of amyloid precursor protein is a major contributor to neuronal death and disease pathogenesis [48]. HT22 mouse hippocampal cells transfected with A*β*_1-42_ displayed a marked increase in apoptosis compared to controls transfected with empty vector, indicated by the presence of condensed and fragmented nuclei (Figure 7(a)). Strikingly, this effect was entirely mitigated by treatment with Immunocal, which preserved neuronal viability to an extent similar to that of empty vector controls (Figure 7(b)).

## 4. Discussion

**Figure 8:**
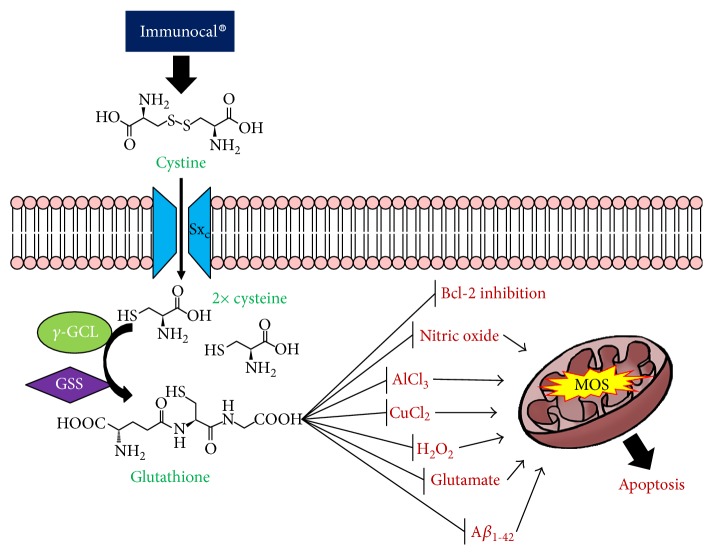
Proposed neuroprotective mechanism of Immunocal. Immunocal provides the essential GSH precursor, cystine, which is transported into cerebellar granule neurons via the system x_c_^−^ antiporter (Sx_c_^−^). Upon entry into the cell, cystine is rapidly hydrolyzed to form two cysteine molecules, which are then utilized in the de novo synthesis of GSH by *γ*-glutamylcysteine ligase (*γ*-GCL) and glutathione synthase (GSS). Newly synthesized glutathione inhibits oxidation caused by a variety of insults, thereby preventing mitochondrial oxidative stress (MOS) and subsequent induction of apoptosis.

Strategies aimed at scavenging ROS, including those that enhance the capacity of endogenous antioxidant defenses like GSH, are actively being investigated as therapeutic approaches for neurodegenerative diseases. In the present study, we assessed the neuroprotective potential of Immunocal, a cystine-rich whey protein supplement, against oxidative stress *in vitro.* This supplement contains high concentrations of proteins such as serum albumin, alpha-lactalbumin, and lactoferrin, which possess a substantial number of cystine residues in the unique nondenatured preparation. In addition, the direct GSH precursor, glutamylcysteine, is also present in the serum albumin fraction of this supplement. Due to these unique features, Immunocal has been used as a cysteine delivery system to boost GSH levels in individuals diagnosed with diseases for which oxidative stress is a prominent underlying factor [31, 32, 49]. Therefore, Immunocal may be an effective approach to elevate GSH in cases of neurodegeneration for which oxidative stress plays a significant role. To this end, we studied the potential of Immunocal to protect neurons *in vitro* from a diverse array of oxidative insults, which are not only known to cause oxidative damage and mitochondrial dysfunction but also to imitate some pathogenic factors in neurodegeneration such as diminished Bcl-2 function, increased levels of nitric oxide, or metal ion toxicity (Figure 8).

GSH depletion is a widely studied phenomenon in cases of neurodegeneration. Although there are multiple mechanisms by which GSH may be depleted, one involves the downregulation of Bcl-2 expression or function. Increased expression of Bcl-2 leads to enhanced GSH synthesis and decreased GSH efflux from the cell [50, 51]. On the other hand, Bcl-2 knockdown leads to decreased levels of tissue GSH [52]. In the current study, we utilized the Bcl-2 inhibitor, HA14-1, to mimic loss of Bcl-2 function and assess the neuroprotective potential of Immunocal. We have previously shown HA14-1 to decrease the cellular GSH pool with a propensity to affect the mitochondrial GSH pool first and induce mitochondrial oxidative stress and intrinsic apoptosis in CGNs [37, 38]. Under these conditions, Immunocal displayed robust neuroprotection, indicating a capacity to counter the effects of mitochondrial GSH depletion and oxidative stress induced by loss of Bcl-2 function. Moreover, the protective effect of Immunocal against Bcl-2 inhibition is dependent upon de novo GSH synthesis as coincubation of Immunocal with BSO blocked neuroprotection.

Another factor implicated in the pathogenesis of several neurodegenerative diseases is copper toxicity. GSH is known to play a significant role in mitigating copper toxicity by facilitating the transport of copper to proteins that can safely store this toxic metal in the intracellular environment [53]. Depletion of GSH disrupts this important process and sensitizes neuronal cells to copper toxicity through copper-associated ROS generation, even when exposed to only trace amounts of copper [17, 54, 55]. Thus, copper toxicity may be a process that is dependent on GSH depletion, and indeed, increased concentrations of copper and dysregulation of copper homeostasis are observed in several neurodegenerative diseases in which GSH status is reduced, including Alzheimer's disease and models of ALS [54, 56]. In our study, elevation of GSH levels in cultured primary neurons with Immunocal proved to be an effective way to ameliorate the toxic effects of copper treatment by attenuating copper-induced lipid peroxidation, resulting in reduced cell death.

Neuroinflammation, in which microglia and astrocytes take on an inflammatory phenotype and secrete toxic factors such as cytokines and nitric oxide, is another major component of neurodegenerative disease [57, 58]. Induction of nitric oxide synthase (NOS) and subsequent production of nitric oxide is a well-established mechanism by which inflammatory cells trigger neuronal cell death [57]. Markers of nitrosative stress are prevalent in tissues from both Parkinson's and Alzheimer's disease patients, indicating a significant role for nitric oxide in disease pathogenesis [59, 60]. Reactive nitrogen species (RNS) such as nitric oxide promote damage to mitochondrial components, leading to dissipation of mitochondrial membrane potential and further increases in ROS and RNS production [42, 43]. This feed forward cycle ultimately exacerbates inflammatory responses and eventually results in neuronal death. GSH is known to detoxify both ROS and RNS, making it an essential antioxidant and key neuroprotective molecule. Consistent with this, preincubation with Immunocal significantly protected CGNs from toxicity induced by the nitric oxide donor SNP.

The neurotoxic effects of aluminum exposure are well documented, and recently, environmental aluminum and aluminum-containing vaccines have garnered attention as potential causes of neurodegeneration. In general, *in vitro* exposure of neural cells to aluminum has been shown to result in pronounced alterations in mitochondrial structure and function, leading to marked increases in ROS, reduction of mitochondrial enzyme activity, and cell death [45]. Aluminum also interferes with the activity of NADP-isocitrate at the mitochondria, decreasing the pool of NADPH that is available and necessary for the regeneration of GSH, and thereby decreasing GSH levels [61]. *In vivo* examination of aluminum neurotoxicity has demonstrated that healthy mice treated with aluminum hydroxide display significant motor deficits and develop pathological features similar to those observed in ALS [62]. These results are notable in that Veterans of the 1990-1991 Gulf War who received vaccines containing aluminum hydroxide adjuvant demonstrate a significant increase in the incidence of ALS, implicating aluminum toxicity as one potential environmental factor in some forms of sporadic ALS [62, 63]. Our experiments clearly demonstrate that Immunocal pretreatment is capable of significantly reducing the degree of neurotoxicity observed with aluminum in CGN cultures. We further confirmed that the protective effects of Immunocal were not due to metal chelation by removing Immunocal-containing media prior to the addition of AlCl_3_.

To determine if the protective action of Immunocal observed in CGNs was reproducible in other neuronal cell types bearing relevance to neurodegenerative disease, we examined the capacity of this supplement to protect NSC34 motor neuron-like cells from oxidative stress and excitotoxicity. NSC34 cells are a hybrid cell line consisting of spinal cord motor neurons fused with mouse neuroblastoma cells [64]. We first analyzed the ability of Immunocal to protect NSC34 cells from H_2_O_2_-induced oxidative stress. Immunocal pretreatment of NSC34 cells dose-dependently attenuated H_2_O_2_-induced cell death. We next examined the potential of Immunocal to ameliorate damage induced by excitotoxic insult in NSC34 cells, which were differentiated by prolonged serum withdrawal to induce the expression of NMDA receptors [47]. Excitotoxicity is known to play a prevalent role in the pathogenesis of multiple neurodegenerative diseases, including ALS, and is intimately linked with both oxidative and nitrosative stress [65]. Immunocal pretreatment of differentiated NSC34 motor neuron-like cells significantly reduced the injurious effects of glutamate excitotoxicity in a dose-dependent manner.

Lastly, we evaluated the ability of Immunocal to defend HT22 mouse hippocampal cells from toxicity induced by the overexpression of A*β*_1-42_. As previously discussed, A*β*_1-42_ is the primary constituent of senile plaques, one of the hallmarks of Alzheimer's disease pathology. In addition, this protein is also known to accumulate with amyloid precursor protein at mitochondria, leading to significant mitochondrial dysfunction [48]. Indeed, A*β*_1-42_ accumulation at the mitochondria has been shown to occur both in transgenic mouse models of the disease and in the brains of Alzheimer's patients [66–68]. Our data indicate that pretreatment with Immunocal was able to preserve HT22 hippocampal cell viability to a significant degree, indicating that GSH supplementation may be an effective way to mitigate cell death caused by A*β*_1-42_-induced toxicity.

## 5. Conclusions

Immunocal was initially studied for application to clinical disorders of immune system deficiency and cancer as an approach to augment the available GSH pool and increase cellular antioxidant and immune system defenses. More recently, Immunocal has been investigated as a potential treatment for disorders involving the CNS. Oral administration of Immunocal for 45 days has been shown to elevate GSH levels in the brains of healthy, nontransgenic mice by up to 300% compared to casein-treated controls, demonstrating that this supplement is able to directly affect the antioxidant status of tissues in the CNS [69]. Furthermore, we recently demonstrated that oral administration of Immunocal in the G93A mutant SOD1 mouse model of ALS delayed disease onset and preserved grip strength to a significant degree, in comparison to untreated transgenic mice [70]. These therapeutic effects correlated with preservation of both blood and spinal cord GSH levels in comparison to untreated transgenic controls, indicating that Immunocal is able to act directly on the CNS to preserve GSH status in the context of neurodegenerative disease. Based on the above studies and the data shown here, we suggest that Immunocal might hold significant potential as a novel therapeutic approach to bolster GSH levels in neurodegenerative disorders for which the underlying pathology involves significant oxidative stress. In the future, it will be of interest to further assess the therapeutic benefit of GSH precursor supplementation with Immunocal in additional preclinical animal models of neurodegeneration and ultimately in clinical trials of patients afflicted with neurodegenerative disorders.

## Figures and Tables

**Table 1:** Immunocal constituents by mass per one packet of supplement (one packet of Immunocal contains approximately 10 g of protein supplement (one serving) in fine powder form and 40 calories per serving).

Component	Supplement content	Percent of total supplement
Whey proteins (*β*-lactoglobulin, immunoglobulin, serum albumin, *α*-lactalbumin, and lactoferrin)	8.8–9.2 g	88–92%
Fat	~0.05 g	<0.5%
Lactose	~0.15 g	<1.5%
Minerals (Ca, Na)	~0.30 g	<3.0%
Moisture	0.5 g	~5%

**Table 2:** Cystine [(Cys)_2_] and glutamylcysteine [Glu-(Cys)_2_] content of Immunocal whey proteins.

Whey protein	Molecular mass (kDa)	Percent of protein fraction	Cystine (Cys)_2_ per molecule	Glu-(Cys)_2_ per molecule
*β*-Lactoglobulin	18,400	56.3%	2	0
Immunoglobulin	166,000	9.2%	4	0
*α*-Lactalbumin	14,200	22.8%	4	0
Serum albumin	66,000	11.1%	17	6
Lactoferrin	77,000	0.7%	17	4

**Table 3:** Cystine [(Cys)_2_] and glutamylcysteine [Glu-(Cys)_2_] content of Immunocal in preincubation culture medium (3.3%, *w*/*v* final concentration).

Whey protein	Total molecules per mL	Total number of (Cys)_2_ per mL	Total number of Glu-(Cys)_2_ per mL
*β*-Lactoglobulin	5.44 × 10^14^	1.09 × 10^15^	0
Immunoglobulin	9.91 × 10^12^	3.96 × 10^13^	0
*α*-Lactalbumin	2.91 × 10^14^	1.17 × 10^15^	0
Serum albumin	3.01 × 10^18^	5.11 × 10^19^	1.80 × 10^19^
Lactoferrin	1.63 × 10^16^	2.76 × 10^17^	6.50 × 10^16^
Final concentration	—	85.3 mM	30.0 mM

## Abbreviations

A*β*: Amyloid beta
ALS: Amyotrophic lateral sclerosis
BH3: Bcl-2 homology domain-3
BSA: Bovine serum albumin
BSO: Buthionine sulfoximine
CGN: Cerebellar granule neuron
DAPI: 4,6-Diamidino-2-phenylindole
*γ*-GCL: *γ*-Glutamyl-cysteine ligase
GSH: Glutathione
GSH-MEE: Glutathione monoethylester
GSS: Glutathione synthase
HA14-1: 2-Amino-6-bromo-*α*-cyano-3-(ethoxycarbonyl)-4H-1-benzopyran-4-acetic acid ethyl ester
ICAL: Immunocal
NAC: N-Acetyl cysteine
NMDA: N-Methyl-D-aspartate
Nrf2: Nuclear factor erythroid 2-related factor-2
ROS: Reactive oxygen species
RNS: Reactive nitrogen species
SOD1: Copper-zinc superoxide dismutase
SNP: Sodium nitroprusside
Sx_c_^−^: System x_c_^−^
TDP-43: TAR DNA binding protein-43.
